# More than the end: OFF response plasticity as a mnemonic signature of a sound’s behavioral salience

**DOI:** 10.3389/fncom.2022.974264

**Published:** 2022-09-06

**Authors:** Dakshitha B. Anandakumar, Robert C. Liu

**Affiliations:** ^1^Wallace H. Coulter Department of Biomedical Engineering, Georgia Institute of Technology and Emory University, Atlanta, GA, United States; ^2^Department of Biology, Emory University, Atlanta, GA, United States; ^3^Center for Translational Social Neuroscience, Emory University, Atlanta, GA, United States

**Keywords:** gap detection, auditory cortex, working memory, echoic memory, plasticity, OFF responses, neural coding

## Abstract

In studying how neural populations in sensory cortex code dynamically varying stimuli to guide behavior, the role of spiking after stimuli have ended has been underappreciated. This is despite growing evidence that such activity can be tuned, experience-and context-dependent and necessary for sensory decisions that play out on a slower timescale. Here we review recent studies, focusing on the auditory modality, demonstrating that this so-called OFF activity can have a more complex temporal structure than the purely phasic firing that has often been interpreted as just marking the end of stimuli. While diverse and still incompletely understood mechanisms are likely involved in generating phasic and tonic OFF firing, more studies point to the continuing post-stimulus activity serving a short-term, stimulus-specific mnemonic function that is enhanced when the stimuli are particularly salient. We summarize these results with a conceptual model highlighting how more neurons within the auditory cortical population fire for longer duration after a sound’s termination during an active behavior and can continue to do so even while passively listening to behaviorally salient stimuli. Overall, these studies increasingly suggest that tonic auditory cortical OFF activity holds an echoic memory of specific, salient sounds to guide behavioral decisions.

## Introduction

We live in a dynamic environment with constantly changing external stimuli that sensory neurons monitor to enable better comprehension of our surroundings. Sensory cues unfolding in time must be integrated, perceived, and differentiated to guide distinct behavioral actions (Stein, 1998; Philiastides and Heekeren, 2009; Mostert et al., 2015; Dunlap and Liu, 2018). For example, one would respond vigilantly to “Fight!” and hurriedly to “Fire!” as the brain processes the diverging spectrotemporal trajectories of these words that start with same phonemes. Updates in stimulus information are reflected in time-varying patterns of neural activity at the sensory periphery, where stimulus onsets and offsets can drive bursts of action potential firing–a topic that has been well-studied (Rose et al., 1967; Meister and Berry, 1999; Wilson and Mainen, 2006; Baden et al., 2011; Peterson and Heil, 2019). At higher-order sensory stations like sensory cortex, much attention has been paid to neural firing while stimuli are “ON,” but new research is revealing the importance of post-stimulus neural activity for distinguishing behaviorally salient cues. Intriguingly, this activity during the “OFF” intervals between stimuli does not just transiently mark signal offsets but reflects how stimulus features unfolding over time carry behavioral meaning–the focus of our review.

The selectivity of temporal patterns of neural activity for the combination of sensory input and behavioral context has long been a part of how ON responses have been characterized, but this has been rarer for OFF responses. For our purposes, “phasic” firing refers to transient excitatory spiking that lasts less than ∼50 ms and is tightly and reliably locked to specific stimulus events across trials, while “tonic” firing refers to a longer-lasting elevated (re. spontaneous) spiking, which is often more variable across trials. Hence, phasic ON responses occurring at stimulus onset can signal its appearance, but they have less ability to signal how the input advances over time (Macknik and Livingstone, 1998; Sugase et al., 1999; Recanzone, 2000; Pei et al., 2009; Joachimsthaler et al., 2014). Meanwhile, tonic ON responses have been shown to indicate stimulus preference and salience and are altered by experience (Bieser et al., 1996; Downar et al., 2003; Wang et al., 2005; Fishman and Steinschneider, 2009). Both phasic and tonic ON responses can occur at short or long latencies from stimulus onset and are associated with distinct functional roles (Lamme et al., 1999; Supèr et al., 2001; Lee et al., 2002; Wang et al., 2005; Yan et al., 2018; O’Sullivan et al., 2020). However, in several situations involving complex stimuli, the behavioral meaning of a signal cannot be fully recognized until stimulus cessation. Whether OFF responses are similarly varied in their temporal profiles and sensitivity to experience is only just beginning to be uncovered.

The accepted role of OFF responses in detecting stimulus termination (He, 2002; Anderson et al., 2016; Awwad et al., 2020; Li et al., 2021), which serves within-and between-channel gap detection, has been reviewed extensively elsewhere (Eggermont, 1999; Phillips, 1999; Elangovan and Stuart, 2008; Weible et al., 2014; Kopp-Scheinpflug et al., 2018). Here we review recent neurophysiological and behavioral studies suggesting that this is only part of its function. To do so, we will first survey some of the coding roles of OFF responses across sensory modalities and then focus on examples from the auditory cortex, for which there has been much recent progress. Specifically, we will discuss distinct sound-driven OFF response patterns and the possible neural mechanisms involved in generating them. Next, we will discuss evidence that OFF responses are not immutable, and while their existence may serve an echoic memory function, their prevalence and time course can nevertheless vary with experience learning about specific stimuli. Intriguingly, this plasticity can linger outside of the learning context and serve as a signature of behavioral salience, even in passive listening, when increases in the pervasiveness and duration of OFF firing may help sustain a stronger echoic memory that supports sound recognition. A re-analysis of data from a published natural learning paradigm further supports this role of tonic OFF responses in reflecting behavioral salience. Lastly, we discuss the possible implications of OFF responses in supporting perceptual decision-making to pave way for future research probing the potential cognitive functions of OFF responses.

## OFF responses across sensory modalities

Separate pathways beginning at the periphery have long been known to help differentiate between the appearance and disappearance of stimuli across a variety of sensory modalities. Newer research is revealing how the OFF pathway conveys other sensory attributes as well. In the visual system, ON-and OFF-responsive neurons originate as early as in the retina and these well-organized pathways remain separate through the lateral geniculate nucleus and up to the visual cortex. ON/OFF neuronal signaling properties arise from push-pull synaptic interactions and are preserved during propagation through the visual pathway (Ferster, 1988; Ryu et al., 2019; Zamarashkina et al., 2020). Together, ON-responsive and OFF-responsive neurons play a key role in enabling better contrast sensitivity and rapid information transfer for both increments and decrements of light intensity (Schiller et al., 1986). OFF responses are essential to indicate changes in visual scenes (Bair et al., 2002), and are modulated by size and spatial frequency (Jansen et al., 2019; Lazar et al., 2021), as well as contrast over a range of luminance (Rahimi-Nasrabadi et al., 2021).

In the somatosensory cortex, OFF responses are thought to result from transient, rapidly adapting fibers and are sensitive to duration of indentation, with their magnitude increasing with longer duration (Sur et al., 1984; Downar et al., 2003; Spackman et al., 2006; Pei et al., 2009; Callier et al., 2019). It has been hypothesized that the interaction between tonically sustained somatosensory cortical responses and phasic stimulus OFF responses could provide better spatial acuity and fine spatial tuning of input stimulus (Bensmaia et al., 2008). However, the sensitivity of OFF responses to stimulus characteristics other than duration has yet to be evaluated.

OFF responses are also common in chemosensory systems (Sato, 1976; Luo and Katz, 2001; Namiki and Kanzaki, 2011; Kato et al., 2014; Devineni et al., 2021). In rodents, odor information is initially encoded in the glomeruli of the olfactory bulb and then integrated in the piriform cortex where a representation of the odor object is first formed (Bolding et al., 2020). There is heterogeneity in odor-evoked response patterns that enable organisms to follow odor trails, with neurons being excited at the onset and offset of stimuli, and some being suppressed during stimuli. Changes in odor concentration at the edges of odor trails are encoded by transient ON and OFF responses, whereas a more stable concentration within the odor plume is encoded by neurons with persistent excitation or suppression (Tantirigama et al., 2017). A similar diversity in odor-evoked responses has been observed in the lateral entorhinal cortex of mice, suggesting that OFF responses may be a general feature of odor-processing circuits (Leitner et al., 2016).

The auditory system is particularly sensitive to temporal changes in stimulus energy emitted by acoustic sources, whose onsets and offsets (relative to the ongoing background sound level) can carry behaviorally important information. Peripheral auditory neurons faithfully encode the fine structure of incoming sound input in their temporal firing pattern to convey perceptual attributes like frequency, duration, and loudness (Johnson, 1998; Joris and Yin, 1998). Auditory OFF responses had been thought to mainly signal the end of a stimulus and help mark silent gaps in sounds. OFF responses appear as early as in the cochlear nucleus (Wickesberg and Oertel, 1990; Ding et al., 1999), and continue to be seen in the superior olivary complex (Dehmel et al., 2002; Kulesza et al., 2003), the inferior colliculus (Kasai et al., 2012), the medial geniculate body (Yu et al., 2004; Bartlett and Wang, 2007; Wang et al., 2008) and the auditory cortex (Recanzone, 2000; Takahashi et al., 2004; Qin et al., 2007; Kato et al., 2015; Baba et al., 2016), where they can vary across cortical subfields, and be particularly amplified (Sołyga and Barkat, 2019; Solyga and Barkat, 2021) and of long duration (Cooke et al., 2020). Interestingly, the time course of auditory cortical OFF response is heterogeneous in ways that can show sensitivity to the precise properties of preceding stimuli, which we expand on in the remainder of this work.

## Diverse temporal firing profiles of auditory cortical OFF responses

Neurons in the auditory periphery and auditory sub-cortical structures can follow rapid changes in acoustic stimuli over 100 Hz, sometimes even as high as 350 Hz (Schuller, 1979; Joris et al., 2004; Nelson and Carney, 2004; Eggermont, 2015). Subsequent stations along the ascending pathway are more limited in their stimulus-synchronized and phase-locked responses to frequency changes in stimuli (Lu et al., 2001; Bartlett and Wang, 2007; Bartlett, 2013). When the sound’s neural fingerprint reaches the auditory cortex, though there are still some synchronized or phasic discharges, rapid acoustic transients are often encoded by non-synchronized or tonic discharges, which integrate the physical acoustic features with more abstract factors such as context, attention, and prior experience with the sound (König, 2005; Budinger et al., 2006; Wang et al., 2008). This mix of firing patterns gives rise to complex temporal response profiles that reflect the sophisticated sound processing occurring in the auditory cortex.

**FIGURE 1:**
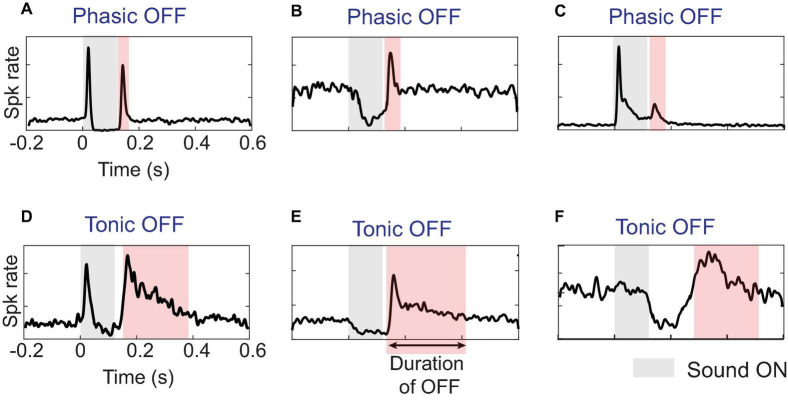
Diverse temporal profiles of OFF responses in single units (SUs): **(A–C)** OFF responses in the auditory cortex can have short duration called phasic response, or **(D–F)** long duration called tonic response. The tonic response can further occur immediately after sound offset, typically within 50 ms or after a long delay. An automated threshold detection algorithm identified the presence of OFF response if activity was greater than three standard deviations above spontaneous activity. Only the activity after the end of sound that was distinct from ON response, either by a dip in response below the threshold or a distinct peak at sound-offset was classified as OFF response.

In general, while a sound is ON, responses can exhibit phasic or tonic excitation, inhibition, or no discernible change from baseline spontaneous firing. Once a sound ends, as illustrated in Figure 1 for various sinusoidally frequency-modulated tones, excitatory OFF responses can appear as elevated firing above the spontaneous rate and are preceded by either a return to baseline or a dip in the ON response. Phasic OFF firing is typically locked with short latency to the end of a sound and shows a peak in firing lasting for less than 50 ms (Figures 1A–C), comparable to phasic ON firing (Heil, 1997; Talwar and Gerstein, 2001; Phillips et al., 2002; DeWeese et al., 2003). While many often assume OFF responses only refer to such phasic firing, the existence and function of tonic OFF firing should not be ignored and may reflect additional mechanisms and functions. Here, we classify OFF responses as tonic when the post-stimulus excitatory firing remains persistently above baseline firing for long durations–sometimes up to a few hundred milliseconds. Importantly, this prolonged activity can at times be itself temporally modulated with brief periods of stronger firing followed by weaker but still elevated firing (e.g., Figures 1D,E). A further subcategory consists of tonic OFF responses that emerge after a delay of anywhere between 50 ms and a few hundred milliseconds (Figure 1F).

Importantly, tonic OFF firing may or may not coexist with ON excitation, though its delineation in the former case requires additional considerations when the ON firing continues up to a sound’s end. If no noticeable dip or peak in firing occurs after sound termination, then whether the post-stimulus tonic activity is just a continuation of sustained ON firing is difficult to judge without a clearer understanding of the underlying mechanisms. If the post-stimulus firing fades away quickly (e.g., ∼15 ms in mice), then it likely is not a distinct OFF response, but rather just reflects the latency for peripheral information about the sound’s end to reach the cortex. Alternatively, if tonic ON firing continues past sound offset for much longer than this, that firing likely engages distinct neural mechanisms than when the sound is ON and could be considered OFF firing even when no obvious dip is observed. However, further studies are needed to fully elucidate the mechanisms that distinguish tonic ON responses from post-stimulus activity.

In fact, given the variable patterns of OFF responses, the underlying cellular and circuit mechanisms generating them are likely diverse. Originally, OFF responses were thought to arise from a post-inhibitory rebound as a neuron is released from long lasting synaptic inhibition. Neurons could have an ON, OFF or both ON and OFF response depending on the timing of activation of inhibitory channels (Takahashi et al., 2004; Hartley et al., 2011).

However, other works have challenged the notion that post-inhibitory rebound is the main mechanism for generating OFF responses, particularly at the level of the auditory cortex. Qin and colleagues argued that not all OFF responses are preceded by suppression in the auditory cortex and instead also result from active spike generation mechanisms involving the integration of synaptic input (Qin et al., 2007). They suggested that ON and OFF responses can be produced by the same neural mechanisms that underlie detection of rapid changes in sound amplitude at both the start and end of a sound. On the other hand, (Scholl et al., 2010) using both *in vivo* whole cell and extracellular recordings concluded that auditory cortical ON and OFF responses often have dissimilar frequency tuning. In observing that an OFF response does not forward suppress a subsequent ON response, they concluded that largely separate sets of thalamocortical presynaptic inputs mediate auditory cortical ON and OFF firing.

One element in common for the three mechanisms discussed above–post-inhibitory rebound, same excitatory presynaptic neurons, or a distinct OFF pathway–is that they focus on phasic OFF firing, which could mainly result from intrinsic and feed-forward synaptic mechanisms. In contrast, tonic OFF responses are potentially generated by a hybrid mechanism that includes a combination of intrinsic cellular and recurrent network dynamics. Typically, slow NMDA receptor currents (Wang, 1999) and dense recurrent connectivity in cortical networks are considered responsible for generating persistent neural activity (Wang, 2001; Brody et al., 2003; Major and Tank, 2004), and this might be applicable to tonic sound-OFF firing as well. Given the diversity in temporal patterns of OFF responses shown in Figure 1, some combination of the phasic and tonic mechanisms (or cellular/synaptic and network mechanisms) are at play in cases when short latency phasic firing transitions into more extended tonic firing (Figures 1 D,E).

Only one study so far has attempted to test this idea through computational modeling of calcium imaging data from mouse auditory cortex. Recurrent connectivity within auditory cortex does help explain OFF responses at a population level, at least in comparison to a simplistic single cell model that assumes stimulus-independent but diverse patterns of excitatory responses of different neurons (Bondanelli et al., 2021). While this is a start, evaluation of mechanisms that specifically drive tonic OFF responses in the auditory cortex awaits further attention, and may require more consideration of network effects, such as re-entrant top-down activity (Ahissar et al., 2009; Sikkens et al., 2019).

## Sound feature sensitivity of OFF responses

**FIGURE 2:**
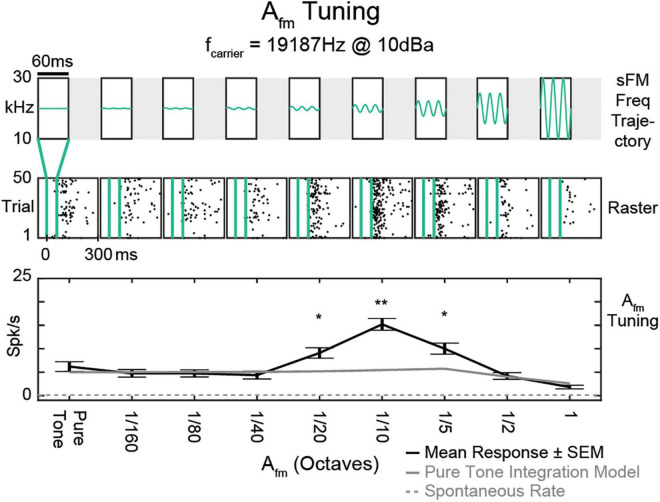
Tuning of OFF response to frequency modulation. A Tuning of an example single unit (SU) to sinusoidally frequency modulated tones centered around its best frequency of 19,187 Hz. Top, Schematic frequency trajectories of each stimulus, with varying frequency excursions (amplitude of frequency modulation, Afm) and all other parameters fixed at: temporal modulation frequency ffm = 50 Hz, center frequency f0 = BF (19,187 Hz), fslope = 0 Hz/s, dur = 60 ms. Middle, Raster responses to stimuli delivered within the vertical green lines. Black dots represent individual spikes. Bottom, Mean response tuning curve (black). Frequency excursions smaller than the typical spectral width of pure tone tuning curves drove better responses than the constant pure tone BF itself. This SU had a peak in Afm tuning at 1/10 octave, with an evoked spike rate more than twice that predicted from just integrating the pure tone excitatory tuning curve over the same spectral range. Larger Afm values reduced firing rates from the peak, which would not be explainable just by its excitatory sensitivity to the brief sound’s static spectrum. Error bars indicate standard error of the mean (SEM). Spontaneous rate (dotted gray line) and rate predicted from integrating the pure tone tuning curve (gray) are also shown. *p, 0.01; **p, 0.0001; Bonferroni-corrected *t* test [adapted from Chong et al. (2020)].

The distinct mechanisms for generating OFF responses discussed above set them up to be sensitive to specific acoustic features beyond just the end of a sound (He, 2001; Liu et al., 2019; Chong et al., 2020). Indeed, OFF responses are often tuned to specific frequencies (Scholl et al., 2010; Sollini et al., 2018), which can be different from a neuron’s tuning during ON responses. Spectrally adjacent ON and OFF receptive fields together can enable direction selectivity for unidirectional frequency modulated sweeps (Tian et al., 2013; Sollini et al., 2018). OFF firing patterns also demonstrate selectivity to the parameters of more complex time-dependent frequency modulations within sounds that have similar overall power spectra (Chong et al., 2020). Post-stimulus firing can be tuned to small frequency excursions around a specific frequency, producing stronger responses than expected from just responding to the spectral content (Figure 2). In addition, the magnitude of sound OFF activity in response to a multi-frequency component sound is altered by changing any single sound frequency component (Lee and Rothschild, 2021; Solyga and Barkat, 2021), further demonstrating the sound feature sensitivity of OFF responses. Thus, as OFF responses begin after stimulus termination, neural firing can be shaped by how an acoustic signal’s spectral content progresses over time up to the sound’s end.

One advantage of such stimulus sensitivity could be for governing the perception and mediating the salience of specific natural sounds, like vocalizations, which have complex spectrotemporal trajectories, often with meanings that can be deciphered only after sound cessation (Wang, 2000; Bar-Yosef et al., 2002; Lewicki, 2002; Seyfarth and Robert, 2003). Additionally, sound tuned OFF neural activity could be a mechanism for the sensory system to maintain a brief echoic memory of the specific preceding stimulus, especially when the OFF response is more tonic or longer lasting (Nees, 2016; Kinukawa et al., 2019). In passive listening marmosets, OFF responses lasting several hundreds of milliseconds long were observed for a wide range of stimuli, with individual neurons selective to specific stimuli (Cooke et al., 2020). The strength of OFF-period activity is not only modulated by acoustic variation within a sound stimulus but also the recent statistical history of sounds when variation from a predictable pattern of stimulation can be highly salient. Indeed, in an oddball paradigm where a repetitive pattern of stimuli was randomly interrupted by a low probability deviant tone, a delayed tonic OFF response selective to the deviant stimulus accounted for true deviance detection (Chen et al., 2015) through a putative predictive coding mechanism (Parras et al., 2017).

## Modulation of OFF responses by behavioral salience

That OFF responses could reflect a mnemonic sensitivity to recent acoustic stimuli, thus contributing to echoic or auditory sensory memory, raises the possibility that they may also be modulated by longer term experiences which make specific stimuli more memorable and salient. Support for this idea comes from several recent studies, including an ethological paradigm wherein the salience of a specific category of short duration (∼60 ms) frequency trajectories is acquired through communication experience. Studies in the maternal mouse acoustic communication model have revealed plasticity in auditory cortex for ultrasonic, single-frequency whistles produced by pups, which gain salience for adult females through engaging in social interactions with pups and performing maternal behaviors (Banerjee and Liu, 2013; Dunlap and Liu, 2018).

**FIGURE 3:**
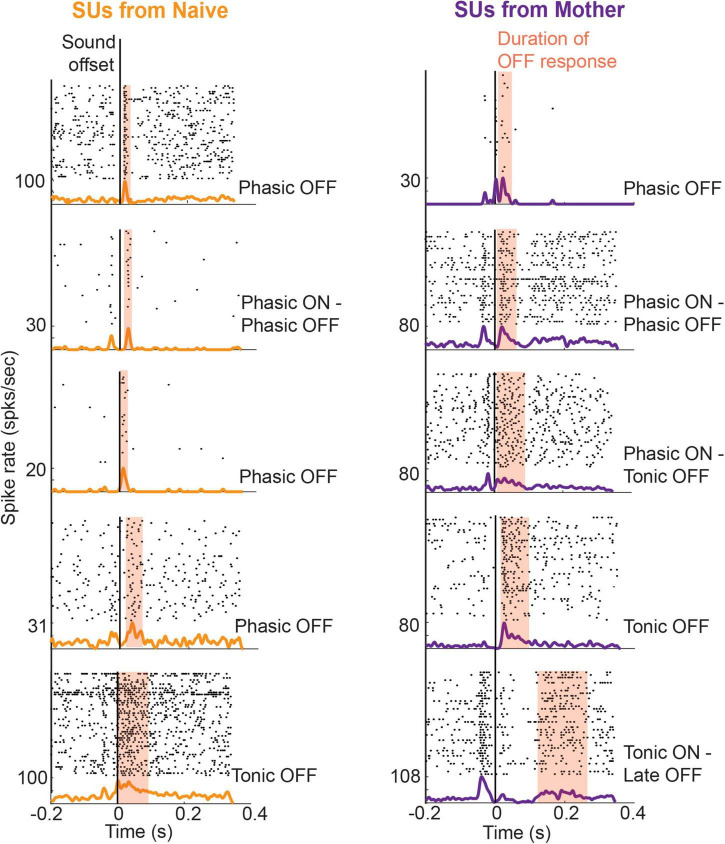
Example single unit (SU) responses from the Core region in mother and naïve animals in response to pup-typical calls [adapted from Chong et al. (2020)]: SUs were recorded from the auditory cortex of passive listening animals to eight different pup calls using single tungsten electrodes. The panels consist of raster and PSTH aligned to the end of a pup call, each of which had durations ranging between 47–60 ms. The duration of OFF responses on a per-call basis was obtained using an automated threshold detection algorithm (MATLAB). OFF firing that lasted for a duration below 50 ms was classified as phasic response. In mothers who had experience caring for pups, OFF responses typically had longer durations falling into the tonic category.

OFF responses to these ultrasonic vocalizations (USV) were observed in both Core (consisting of the main cortical targets of the lemniscal thalamocortical pathway, i.e., primary auditory field, anterior auditory field, and ultrasound field) and secondary (A2) auditory cortex of female mice listening passively to these sounds, regardless of whether they had cared for pups (Figure 3). Following pup care experience though, maternal mice had significantly more neurons with an OFF response for pup vocalizations in A2 compared to naïve virgin mice, while the strength of A2 ON responses weakened. Furthermore, the tuning of A2 OFF responses shifted toward frequency trajectory parameters that enhance the discrimination of pup USVs from another category of less salient USVs (Shepard et al., 2015; Chong et al., 2020). These changes in the sound-OFF activity represent an alternative mechanism of experience-dependent plasticity from the usual tonotopic map expansion (Shepard et al., 2013), which was absent in this ethological case (Shepard et al., 2016). The ability of OFF responses to influence categorical learning of perceptually important sounds has also been observed in Mongolian gerbils that were trained to learn categories of rising and falling frequency modulated tones. An ON component of electrocorticographical recording, locked to stimulus onset, was observed in both trained and untrained animals. However, a late OFF response occurring a few seconds beyond stimulus offset was unique to trained animals (Ohl et al., 2001).

**FIGURE 4:**
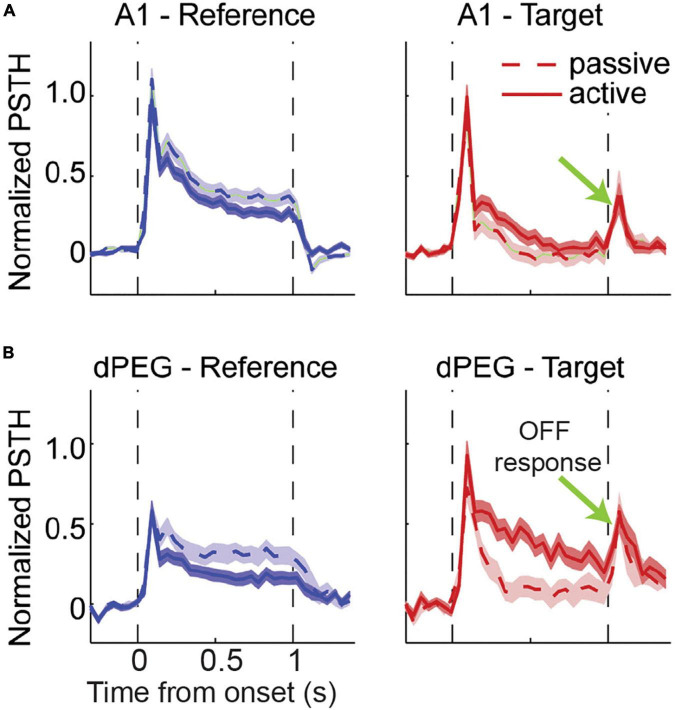
Comparison of average response to target and reference tone across hierarchical auditory regions. Ferrets were conditioned to freely lick during the presence of reference stimuli (broadband rippled noise or narrow band noise) until they heard a pure tone target to avoid mild tail shock. Animals were trained to stop licking soon after target tone ended for a minimum of 400 ms. **(A)** Response to reference and target in Core auditory cortex. The magnitude of evoked ON response for were comparable for the reference and target signal. Surprisingly, OFF response is unique to the target and present in both passive and active cases. **(B)** Response to reference and target in secondary auditory cortex-dorsal posterior ectosylvian gyrus (dPEG). OFF response remained unique to target signal and absent for reference stimuli and comparable between passive listening and behaving animals [adapted from Atiani et al. (2014)].

In several other non-ethological paradigms where animals are trained to associate a specific “target” stimulus to a behavioral response while ignoring “reference” sounds, OFF responses demonstrate selectivity to the acoustic features and reflect the behavioral salience of the preceding sound signal. Associative learning of continuous sound sequences in mice produced long-lasting enhancement of persistent OFF activity to the trained sound and a significant shift from ON to OFF responses in the primary auditory cortex (Lee and Rothschild, 2021). In ferrets trained to cease licking from a waterspout upon hearing a target sound within a sequence of safe reference sounds (Figure 4), ON firing was comparable between the reference and target sounds, but only the target elicited an OFF response of a few hundred milliseconds duration (Atiani et al., 2014). Beyond Core auditory regions, OFF responses for the target can be further amplified in secondary and tertiary areas to improve the contrast between task-relevant categories, and potentially contribute to downstream working memory and motor control (Atiani et al., 2014; Elgueda et al., 2019). Such hierarchical fields within the auditory cortex are positioned to extract behaviorally relevant information from an incoming sound input and optimally transfer it to higher order brain areas in order to act on these signals, and sensory OFF responses may be essential for this, as we discuss next.

## Mnemonic function of OFF responses for behavioral tasks

The patterns of firing by sensory cortical neurons can be swiftly modulated in response to changes in external stimulus events and carry significant information on the timescale of milliseconds (Buračas et al., 1998; Kayser et al., 2010; Runyan et al., 2017; Stigliani et al., 2017; Cavanagh et al., 2020). However, perceptual decisions that lead to behavioral actions involve recognizing, integrating, retaining, and consolidating sensory evidence over longer timescales (Stein, 1998; Shadlen and Newsome, 2001; Andersen and Cui, 2009; Hernandez et al., 2010; Honey et al., 2012; Waskom and Kiani, 2018) and across modalities (Franzen et al., 2020), sometimes even in the absence of any continuing physical stimulation. Hence, in the many cases when cues are used to inform subsequent actions, the ability of stimuli to modulate behaviors on a timescale slower than the stimulus itself is important for navigating the sensory world, and stimulus-specific, sensory cortical OFF responses are well-positioned to aid in this process.

Typically, studies probing the role of sensory areas in supporting perceptual decision-making require subjects to evaluate a sequence of stimuli separated by a delay and then respond during an interval when the stimulus itself is absent. In the auditory domain, researchers have documented heightened auditory cortical firing after the end of a stimulus used to cue a later behavioral task (Scott et al., 2014; Huang et al., 2016). For example, when monkeys were trained to discriminate between tones separated by a one second delay, a substantial portion of auditory cortical units in behaving animals showed elevated firing relative to passive listening throughout the delay period (Gottlieb et al., 1989). Increased OFF firing during such short delays may indeed serve an echoic memory function. This was also apparent in another monkey study, where there was enhanced OFF activity at the beginning of a longer 5 s delay between tone pairs (Bigelow et al., 2014). Retaining and rehearsing stimulus information for longer durations might contribute to working memory mechanisms, thus hinting at another role of OFF responses (Cowan et al., 2005; Unsworth and Engle, 2007; Aben et al., 2012). These effects are not exclusive to primates, as heightened OFF firing is also seen during similar tone pair discrimination tasks in rats (Sakurai, 1990; Shinba et al., 1995) and mice (Yu et al., 2021).

A causal role for such auditory cortical OFF activity in behavioral decisions becomes apparent from studies that externally manipulate post-stimulus firing. As would be expected, performance was impaired when OFF activity in the auditory cortex was optogenetically silenced during sound-feature detection tasks that traditionally rely on phasic OFF responses, such as sound duration estimation (Li et al., 2021). Importantly though, even in tasks where OFF responses are not needed *per se* to explicitly encode the relevant sound feature, their presence nevertheless facilitates the ability to do the task well. In mice conducting a delayed match-to-sample tone discrimination task with < 2 s delay, inhibiting neural firing during an early post-stimulus period (< 800 ms) worsened performance (Yu et al., 2021). Interestingly though, doing so late in the delay (> 800 ms) did not, implying that the mnemonic role relevant for behavior may have been transferred to other brain areas by that point.

**FIGURE 5:**
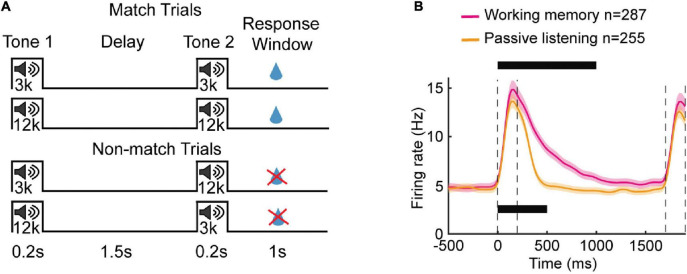
OFF-period activity in auditory cortex helps retain working memory. **(A)** Schematic of working memory task. Mice were trained to compare between a sample tone (3 kHz or 12 kHz) and test tone (3 kHz or 12 kHz) of 0.2 s that were separated by a delay period of 1.5 s. The animals were rewarded with water if they licked during the 1 s response window in matched trials i.e., trials with sample and test tone of same frequency. **(B)** Average population PSTH of neurons recorded during working memory task (*n* = 287) and passive listening paradigm (*n* = 255), both recorded in well-trained mice. The bars on the top and bottom indicate successive 100 ms bins in which the firing rate was above a threshold, for working memory and passive listening cases, respectively. Time 0 is the onset of the first tone. The shaded area represents S.E.M and dashed vertical lines indicate stimulus duration. The duration of sound-OFF response was tonic in nature for both active and passive cases; however, task engagement resulted in longer OFF activity [figures adapted from Yu et al. (2021)].

Notably, stimulus-specific OFF responses can be seen even when there are no behavioral actions required and the sounds do not have any relevance (Chong et al., 2020; Cooke et al., 2020). These “passive listening” responses do not require stimuli to be particularly meaningful in terms of being specifically associated with an action (Cooke et al., 2020), though when they are, OFF responses can become more prominent (Gottlieb et al., 1989; Atiani et al., 2014; Chong et al., 2020). There are differences though between OFF responses during active responding versus passive listening conditions. For example, more neurons tend to show OFF responses in active behavior (Gottlieb et al., 1989), and when the task requires a delay, the duration of tonic OFF firing can extend longer into that delay period when performing the task compared to when animals are just listening passively (Yu et al., 2021). Intriguingly, the duration of OFF responses in the passive case can still last many hundreds of milliseconds (Figure 5)–longer than might be expected just from phasic OFF firing–raising the possibility that these changes in OFF duration provide a signature of learning behaviorally relevant stimuli that persists even outside the context of behavior.

**FIGURE 6:**
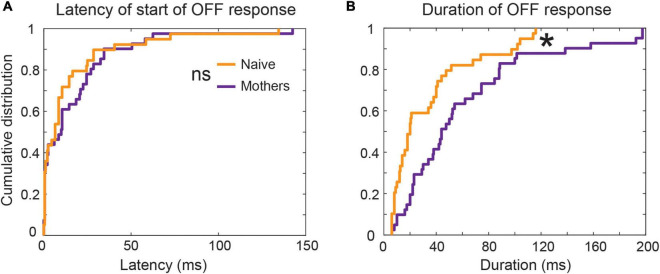
Latency and duration of OFF response on a per-call basis in naïve and mother mice: Among SUs tuned to frequency range of USVs i.e., between 45 and 80 kHz, 16 SUs in mothers and 19 SUs in naïve animals had an OFF response. Further, there were 46 distinct combinations of SU*call in mothers and 34 SU*call in naive on per-call basis analysis. The start of OFF response was estimated by the threshold detection algorithm as the time at which firing rate crossed three standard deviations above the mean spontaneous activity. **(A)** The latency of start of OFF response from stimulus termination was comparable between mothers and naives (*p* > 0.05, KS test). **(B)** Mothers had significantly longer per-call OFF neural activity in response to pup-USVs compared to pup-naïve animals. *p < 0.05, KS test. This result remained consistent when the mean duration of post-stimulus activity across responsive calls per-SU was computed [figure re-analyzed data from Chong et al. (2020)].

To examine that possibility further, we looked back at data from Chong et al. (2020) to analyze the duration of auditory cortical OFF responses recorded from passive listening female mice that found pup USVs behaviorally salient (e.g., mothers) or not (e.g., naïve virgins). We saw that the duration of the OFF response was significantly higher in mothers, with more cases of long tonic OFF responses (Figure 6). Meanwhile, OFF responses in naïve animals were predominantly phasic, though no differences were seen in OFF response latencies. These results held whether we considered both Core and A2 together, or only looked at Core units. Both phasic and transient OFF responses appeared to be sensitive to the specific acoustic features within calls, since only a select number of calls per single unit gave rise to an excitatory response at sound termination. Our analysis expands on our earlier finding of an increase in prevalence of OFF responses in A2 (Chong et al., 2020), to now also show a shift across auditory cortical fields from phasic to more tonic OFF firing in mothers as the USVs gained behavioral significance–even though they were only listening passively to the calls.

## Linking rapid sensory processing to timescales of behavioral decisions

**FIGURE 7:**
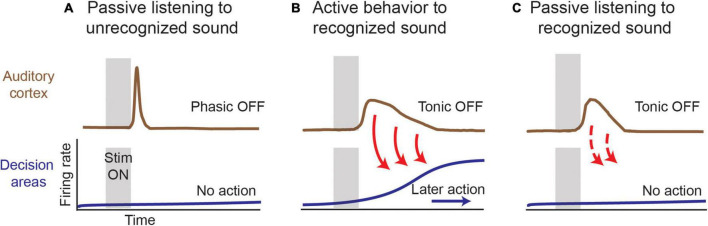
Model for role of OFF responses in reflecting stimulus salience. **(A)** An unrecognized or behaviorally irrelevant sound can elicit OFF responses that are phasic in nature and do not trigger downstream mechanisms that might activate working memory and decision circuits. **(B)** In an auditory sound cued task, a behaviorally relevant stimulus gives rise to long duration tonic OFF responses that might feed into downstream networks to help facilitate echoic or working memory retention, depending on the task complexity. **(C)** In passive listening conditions, tonic OFF responses to a behaviorally meaningful sound to persist but are characterized by shorter durations in comparison to **(B)**. At the level of sensory cortex, a recognized sound continues to elicit differential OFF response that can feed forward to downstream areas irrespective of an impending behavior. The absence of an extended duration could reflect top-down modulation that is exclusive for cases where an action or behavior is involved.

The results reviewed above suggest the conceptual model shown in Figure 7 for a potential function of auditory cortical OFF firing in linking the sensory representation of recognized sounds carried by stimulus-specific subpopulations of neurons to behavioral actions that are performed on a slower time scale. A variety of stimuli can elicit OFF responses, even when individuals are simply listening to meaningless sounds. In that case, the OFF responses are predominantly phasic or short duration in nature and may serve as a brief echoic memory trace (Cooke et al., 2020). As a sound cue gains behavioral salience for triggering decisions and actions on a longer time scale, the stimulus-specific auditory cortical OFF responses become more tonic, lengthening in duration to extend into delay periods between stimuli and actions. We hypothesize that this activity seeds circuits that mediate working memory in sound-driven behaviors. Finally, once this sound cue’s meaning has been acquired, evoked tonic OFF responses remain long in duration, even outside of the behavioral context, which we interpret as a mark of stimulus recognition irrespective of whether or not an impending action ensues. Thus, in our conceptual model, OFF responses are a substrate for echoic memories, and when a sound is more meaningful and actionable, their prevalence and duration increase so that the echoic memory can be used to guide behavior.

How might these differences in sensory cortical OFF firing affect perceptual decisions? Neural models of decision-making generally assume that stimuli generate discrete boluses of sensorineural input, which are then integrated over time to accumulate evidence toward a decision threshold (Brown and Heathcote, 2008; Brunton et al., 2013; Ratcliff and Rouder, 2016). The intrinsic timescales for integration depend on where in the cortical hierarchy one looks, with short timescales on the order of tens of milliseconds in sensory regions extending to hundreds of milliseconds in prefrontal cortical areas (Murray et al., 2014). In fact, network models that replicate this temporal hierarchy (Chaudhuri et al., 2015; Li and Wang, 2022) find that even if the sensory cortical evoked response is brief, that impulse still generates extended periods of firing in higher-order regions. If behavioral salience leads to more sensory cortical neurons with longer windows over which they inject their neural bolus into downstream evidence accumulation circuits, then decision thresholds might be reached earlier, and evoked activity could be sustained longer. Hence, OFF response plasticity for specific salient cues may be a way for prior experience to prime the sensory system’s contribution to making perceptual decisions quickly and to bridging sensorineural activity to later and slower behavioral actions.

## Conclusion and future perspectives

The studies discussed in this review reveal how OFF responses can encode more than just a sound’s termination. They are sensitive to a sound’s acoustic features and can be markers of its behavioral significance while helping to maintain stimulus-specific information as a brief memory trace useable for later behavioral actions. Akin to its ON counterpart, the temporal profile of OFF firing plays a role in dictating its functional significance. While phasic OFF responses that are tightly locked to stimulus termination might be important indicators of sound cessation or gaps, tonic activity can potentially be neural correlates of learned sound recognition. A presumed goal of auditory neural representations is to enable efficient coding of sensory evidence in a way that highlights its behavioral salience when the information is propagated downstream, even in the absence of an impending behavior. Thus, OFF responses continue to persist in passive listening animals. The heterogeneity in neuronal responses at the level of the auditory cortex confers with the ability to integrate information about physical acoustics of the sound with contextual information necessary for encoding perceptual salience.

The ability of OFF responses to encode stimulus salience opens new avenues for delineating how sensory representations are translated into neural constructs of perceptual decision-making. Tonic OFF responses could help mediate between sensory and association areas which inherently process information on different timescales. Sensory stimuli change at fast timescales and often arrive in long streams with gaps in between. It is, however, essential to evaluate the sequence of inputs and integrate sensory evidence over time before making a judgment. This can lead to a disparity between timescales of response in sensory cortices and downstream areas. In order to facilitate efficient transfer of task-related information to higher order areas with slower dynamics, sensory neurons must represent not only the beginning of an input but also keep monitoring for changes during a long stream of stimulus and hold on to that information in the presence of any gap between stimulus bouts. The emergence of OFF-period activity locked to a sound’s end but prolonged in firing could be a possible neural mechanism to ensure that sensory systems can encode the precise timing of rapid sensory stimulus changes to modulate slow timescale decisions.

## Author contributions

DA researched the literature and wrote a first draft of the manuscript. RL wrote sections of the manuscript. Both authors contributed to the conception and execution of this review article, outlined the sections, edited the manuscript, contributed to the manuscript revision, read, and approved the submitted version.
